# Changes in Chlorophyll *a* Fluorescence in *Ipomoea batatas* (Convolvulaceae) Genotypes Under Attack by *Bedellia somnulentella* (Lepidoptera: Bedelliidae)

**DOI:** 10.3390/plants14223529

**Published:** 2025-11-19

**Authors:** Maria J. S. Cabral, Rodrigo A. Pinheiro, Isabel M. Silva, William S. B. Ngamgna, Marcio Schmiele, Germano L. Demolin Leite, Muhammad Haseeb, Marcus A. Soares

**Affiliations:** 1 Center for Biological Control, Florida Agricultural and Mechanical University (FAMU), Tallahassee, FL 32307-4100, USA; jessica1.cabral@famu.edu (M.J.S.C.); muhammad.haseeb@famu.edu (M.H.); 2 Departamento de Agronomia, Universidade Federal dos Vales do Jequitinhonha e Mucuri (UFVJM), Diamantina 39100-000, MG, Brazil; bio.rodrigopinheiro@gmail.com (R.A.P.); ibelmoreira@yahoo.com.br (I.M.S.); bitbeu.ngamgna@ufvjm.edu.br (W.S.B.N.); marcio.sc@ict.ufvjm.edu.br (M.S.); 3 Instituto de Ciências Agrárias, Universidade Federal de Minas Gerais (UFMG), Montes Claros 39404-547, MG, Brazil; germano.demolin@gmail.com

**Keywords:** chlorophyll, biological stress, leaf miners, sweet potato

## Abstract

Sweet potato, *Ipomoea batatas* (L.) Lam., is a major food crop in developing countries and is considered essential for the food security of low-income populations. Among the factors limiting its production is the pest *Bedellia somnulentella* (Zeller) (Lepidoptera: Bedelliidae), recently recorded in Brazil, and for which no chemical control methods are available. Therefore, understanding the physiological changes caused by this pest attack is important to support management strategies. The objective was to evaluate chlorophyll *a* fluorescence parameters in eleven sweet potato genotypes subjected or not to *B. somnulentella* attack. A completely randomized design (CRD) was used, consisting of eleven treatments and twenty replicates. Initial fluorescence (F_0_), maximum fluorescence (Fm), maximum/minimum fluorescence (Fm/F_0_), variable fluorescence/maximum fluorescence ratio (Fv/Fm), and electron transport rate (ETR) were analyzed. In most of the genotypes evaluated, a reduction in F_0_ was observed under attack by *B. somnulentella*, indicating photosynthetic stress, except in genotypes UFVJM 08, 18 and 291. Non-injured plants presented Fv/Fm values between 0.51 and 0.75, while attacked genotypes exhibited reduced values (0.35 to 0.53). ETR was also lower in damaged plants, evidencing photosynthetic stress. The results demonstrated a negative correlation between Fm/F_0,_ Fv/Fm and F_0_. UFVJM 08, UFVJM 18, UFVJM 291, Brazlândia branca and Rubissol genotypes exhibited greater stability, maintaining more balanced fluorescence responses under pest pressure. Physiological differences between genotypes may reflect agronomic responses in the field.

## 1. Introduction

Sweet potato, *Ipomoea batatas* (L.) Lam. (Convolvulaceae) is a crop of great economic, nutritional and social relevance [1], considered the seventh most important cultivated plant in the world [2,3]. Its consumption contributes to food and nutritional security, as it is widely used in both human and animal nutrition [4]. Furthermore, sweet potato has high adaptability to different soil and climate conditions, possessing relative resistance to environmental stresses and a good response to sustainable management [5,6]. In the agricultural context, its short cycle and the possibility of multiple annual harvests favor income generation for small and medium-sized producers.

In this way, sweet potatoes play a strategic role in agricultural development and in promoting more resilient and sustainable food systems. However, pests represent one of the primary challenges to this crop production, compromising roots productivity and quality [7,8].

The moth *Bedellia somnulentella* (Zeller) (Lepidoptera: Bedelliidae) is a leafminer pest of economic importance to sweet potatoes [9]. Its larvae feed on the mesophyll of leaves, forming serpentine mines that impair leaf function and overall plant development [10]. In severe infestations, intense defoliation and stress can occur, leading to reduced root growth and decreased productivity [9]. The presence of leafmining insects in crops can directly compromise the photosynthetic capacity of host plants. By feeding on the leaf mesophyll, these insects damage essential photosynthetic tissues, reducing the functional area of the leaves [11,12]. This relationship between herbivory and plant physiological performance is relevant in crops such as sweet potato, where leaf losses can negatively affect the accumulation of reserves in the roots [13,14]. One of the mechanisms affected is the efficiency of electron transport by photosystem II (PSII), resulting in changes in chlorophyll *a* fluorescence emission [15].

Despite the global importance of sweet potatoes, there is a lack of information on their physiological responses to infestation by leafminers such as *B. somnulentella*. Furthermore, as it is a recently recorded pest in Brazil, few studies have addressed its physiological impacts and the damage it causes to crops [10]. Chlorophyll fluorescence is a rapid and non-destructive tool for early detection of photosynthetic changes caused by biotic stresses [13,14,16].

This study aimed to evaluate the effects of *B. somnulentella* infestation on chlorophyll *a* fluorescence parameter in different sweet potato genotypes, seeking to identify patterns of physiological tolerance. The results reveal new insights into genotype-specific photochemical responses, which contribute to the development of resistance selection strategies in breeding programs.

## 2. Results

In all evaluated genotypes, a reduction in F_0_ was observed under *B. somnulentella* attack, indicating photosynthetic stress and lower efficiency in initial energy capture. This decrease was more evident in UFVJM 01, UFVJM 02, UFMG 03, UFVJM 04, UFVJM 91, UFVJM 526, Brazlândia branca, and Rubissol, in which the differences between treatments were statistically significant (*p* ≤ 0.05) (Table 1).

A reduction in Fm was detected in four genotypes (UFVJM 04, UFVJM 18, UFVJM 291, and UFVJM 526), suggesting that these genotypes have a greater sensitivity in Fm to pest attack, indicating a reduced capacity of photosystem II to reach its maximum fluorescence (Table 1).

For the Fm/F_0_ ratio, higher values were found in non-attacked plants for all genotypes evaluated (Table 1). Plants with Fm/F_0_ values ranging from 3 to 6 do not present stress in the photosynthetic apparatus, indicating that the electron transport chain is functioning properly.

The Fv/Fm parameter, considered a primary indicator of photosynthetic efficiency, displayed marked reductions in genotypes under attack, with significant differences in UFVJM 01, UFVJM 02, UFMG 03, UFVJM 04, and UFVJM 91, revealing that these genotypes undergo substantial losses in PSII efficiency when under *B. somnulentella* pressure. Values in attacked plants ranged from 0.35 to 0.53, while non-attacked plants maintained higher levels (0.64–0.75) (Table 1).

Lower ETR values were observed in UFVJM 2 and UFVJM 526 genotypes exposed to *B. somnulentella*. These results indicate that most of the genotypes tested tend to maintain electron flow even under biotic stress, but with variable physiological resilience among them.

The UFVJM 08, UFVJM 18, UFVJM 291, Brazlândia Branca, and Rubissol genotypes showed greater overall stability, maintaining more balanced fluorescence responses (differences in up to two parameters) under pest pressure.

**Figure 1:**
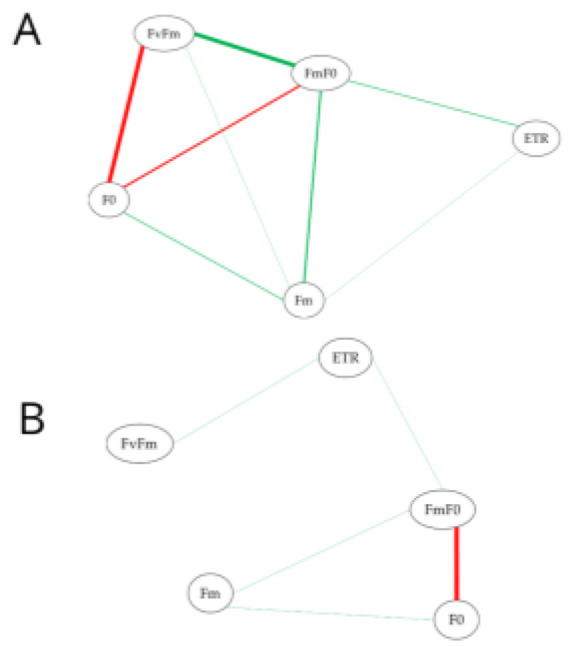
Network structures estimated based on Spearman’s correlation (*p* < 0.05) for leaves of *Ipomoea batatas* attacked (**A**) and not attacked (**B**) by *Bedellia somnulentella*. Each node represents a chlorophyll fluorescence variable: initial fluorescence (F_0_), maximum fluorescence (Fm), ratio between variable and maximum fluorescence (Fv/Fm), ratio between maximum and initial fluorescence (Fm/F_0_), and electron transport rate (ETR). The color of the lines denotes positive (green) or negative (red) correlations, and their thickness represents the magnitude of the correlation coefficient.

Among the attacked plants, a strong positive correlation was observed between the variables Fv/Fm and Fm/F_0_ and a strong negative correlation between the variables Fv/Fm and F_0_ (Figure 1A). When estimating the Spearman correlation for the non-attacked plants, a strong negative correlation was observed between Fm/F_0_ and F_0._ For the other variables related to chlorophyll *a* fluorescence, a weak correlation was observed between the variables (Figure 1A,B).

**Figure 2:**
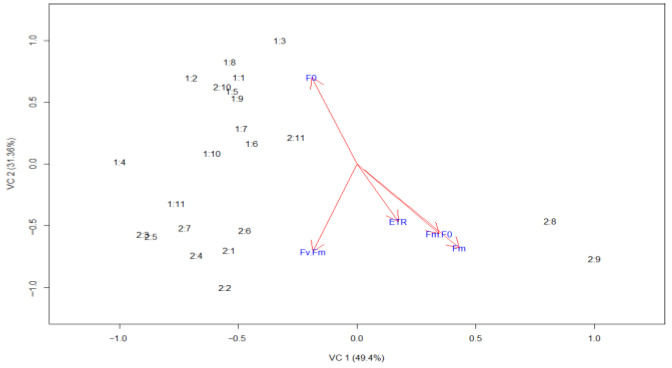
Canonical variable analysis (PCA) of *Ipomoea batatas* genotypes not attacked (group 1) and attacked (group 2) by *Bedellia somnulentella.* Each point represents a genotype, and the arrows indicate the contribution of each chlorophyll fluorescence variable to group discrimination. The first canonical axis (Can1 = 49.4%) is associated with photosystem II efficiency (Fv/Fm, Fm/F_0_, ETR), while the second axis (Can2 = 31.36%) corresponds to basal fluorescence (F_0_). Genotypes are ordered as follows: (1) UFVJM 01, (2) UFVJM 02, (3) UFMG 03, (4) UFVJM 04, (5) UFVJM 08, (6) UFVJM 18, (7) UFVJM 91, (8) UFVJM 291, (9) UFVJM 526, (10) Brazlândia branca, and (11) Rubissol.

The scatterplot differentiates the non-attacked genotypes (group 1:1:1; 1:2–1:11), which are predominantly distributed above the *Y*-axis, from the attacked genotypes (group 2:2:1; 2:2–2:11), which are mainly positioned below the *X*-axis. This behavior indicates marked physiological differences between plants attacked and those not attacked by the pest. Arrows in the same direction indicate a positive correlation between the variables. Thus, the variables Fv/Fm, ETR, Fm/F_0_, and Fm exhibit a positive correlation with each other (Figure 2).

## 3. Discussion

Chlorophyll fluorescence parameters, such as F_0_, Fm, Fv, and ETR, are important for assessing the efficiency of photosystem II (PSII) and the overall health of the plant’s photosynthetic apparatus [12,15,17]. Plants attacked by pests often experience changes in these parameters, reflecting stress and damage. *Bedellia somnulentella* consumes photosynthetically active tissues (chlorophyll parenchyma, palisade, and spongy parenchyma), which disrupts the transport of photoassimilates. However, the response of fluorescence parameters can vary depending on plant resistance to the pest, as seen in resistant versus susceptible cultivars [17,18,19]. Thus, resistance and susceptibility are often linked to the plant’s ability to maintain photosynthetic efficiency and manage stress-induced damage [19]. Understanding these dynamics enables the selection of specific genotypes and the development of effective pest management strategies, thereby improving plant resistance.

The initial chlorophyll *a* fluorescence (F_0_) represents the minimum fluorescence yield when all PSII reaction centers are open and the quinone QA is fully oxidized [20,21,22]. Changes in this parameter may indicate structural damage to the photosystem II reaction centers or impaired energy transport from the antenna complexes to the photosystem I and II reaction centers [19,23,24]. The increase in F_0_ in sweet potato plants attacked by *B. somnulentella* may indicate that the PSII reaction centers are not fully open, resulting in electron accumulation due to a lack of energy transport and, consequently, damage to the photosynthetic system [20]. Therefore, the increase in F_0_ may result in a reduction in photosystem II efficiency (lower Fv/Fm) and a decrease in the electron transport rate (ETR). In corn genotypes attacked by *Spodoptera frugiperda*, J. E. Smith (Lepidoptera: Noctuidae) also observed an increase in F_0_ [12]. The rise in F_0_ in agricultural crops can serve as an early indicator of stress before visible symptoms appear, allowing intervention and management strategies [25,26]. Although F_0_ levels were not affected in the UFVJM 08, UFVJM 18 and UFVJM 291 genotypes attacked by *B. somnulentella*, Fv/Fm values below three are already indicative of reduced photosynthetic efficiency in these plants [27,28]. Because sweet potatoes are tuberous root crops with source-sink dynamics, foliar damage that reduces photosynthetic capacity can affect the allocation of assimilates to storage roots and, consequently, the final yield. Leaf miner damage, caused by *Liriomyza trifolii* (Burgess) (Diptera: Agromyzidae), did not reduce the photosynthetic rates of the remaining leaf tissue in potato plants, despite inducing stress reflected in fluorescence measurements [29]. This suggests that insect damage may not directly affect chlorophyll *a* levels, but impact the overall physiological health of the plant, potentially leading to premature leaf senescence and a gradual reduction in canopy photosynthesis over time [29].

Fv/Fm is directly related to the energy of photosynthesis and the energy lost in the form of heat and fluorescence under plant stress conditions [30,31]. *Liriomyza trifolii* damage altered the photochemical efficiency of potato leaves, as indicated by an increased Fv/Fm ratio in infested leaflets compared to controls, reflecting a physiological response to leaf miner injury [29]. Leaf mining by *Phyllocnistis citrella* Stainton (Lepidoptera: Gracillariidae) larvae reduced photosynthetic and gas exchange parameters in all citrus cultivars tested, with a substantial decrease in Fv/Fm values [32]. Leaf mining pests feed on photosynthetic active substances, sap, nutrients and the structures involved in gas exchange, reducing the highest variable Fv/Fm ratio [18]. This parameter decreased in the genotypes attacked by *B. somnulentella*, within the range of 0.35 and 0.53. This is due to the consumption of the leaf mesophyll, which consequently reflects a reduction in photosynthetic efficiency and possible damage to the plant’s physiological state [12,33]. The Fm/F_0_ ratio, another indicator of the photochemical efficiency of photosystem II, exhibited marked reductions across all sweet potato genotypes under *B. somnulentella* attack, revealing considerable heterogeneity in their responses. In general, reductions exceeded 50% compared to the control, indicating a substantial decline in the capacity of photosystem II to achieve maximum fluorescence relative to minimum fluorescence, thereby reflecting photosynthetic stress [20,34].

Maximum fluorescence (Fm) occurs when QA, the main electron acceptor of photosystem II, is completely reduced [21,22]. This phenomenon results from the application of a saturating light pulse, which reduces quantum yield by promoting the closure of the photosynthetic system’s reaction centers, culminating in maximum fluorescence emission (Fm) [35,36]. Thus, sweet potato genotypes with reduced Fm suggest a reduced ability of PSII to efficiently utilize light energy due to a deficiency in quinone A (QA) photoreduction [35,36,37]. This deficiency may be associated with the inactivation of photosystem II in the thylakoid membranes, directly affecting the electron flow between photosystem II and I [20,34,36]. The type of pest, the plant species and the specific resistance mechanisms of the cultivar may influence the relationship between Fm and pest attack. For example, resistant cultivars may show less pronounced changes in Fm compared to susceptible ones [18,19]. Susceptible barley and wheat cultivars infested with aphids showed lower Fm values, a result similar to that observed for sweet potato cultivars, where changes varied according to genotype, with only UFVJM 04, 18, 291, and 526 showing a reduction in this parameter.

The electron transport rate (ETR) of genotypes attacked and not attacked by *B. somnulentella* differed only in genotypes UFVJM 02 and 526. ETR is directly associated with the overall photosynthetic performance [18,38]. The estimated electron transport rate may have a drop in quantum yield when a plant is under herbivory [39]. In *Brassica oleracea* (Brassicaceae) and *Phaseolus vulgaris* (Fabaceae) plants attacked by *Murgantia histrionica* Hahn and *Nezara viridula* Linnaeus (Hemiptera: Pentatomidae) damage to electron transport was observed, affecting the production of ATP, NADPH and impairing the photosynthetic process [40,41]. In an evaluation with *Solanum lycopersicum* (Solanaceae) and *Gossypium hirsutum* L. (Malvaceae) plants with high infestation of *Phenacoccus solenopsis* Tinsley (Hemiptera: Pseudococcidae), the authors reported a lower electron transport rate [42]. Many leafminer species can reduce the net photosynthesis of host crops by damaging leaf tissue [11,43]. The lower ETR values in the genotypes attacked by *B. somnulentella* are due to the consumption of the leaf surface, reducing the active photosynthetic area and the production of photoassimilates, causing an imbalance between the capture and use of excitation energy, resulting in a negative relationship and, consequently, high physiological stress in these plants [12].

The canonical variable analysis demonstrated a clear separation between non-attacked and attacked genotypes, confirming that pest pressure induced distinct physiological responses among sweet potato genotypes. The first canonical axis (Can1), which explained 49.4% of the total variance, was mainly associated with the photosystem II efficiency parameters Fv/Fm, Fm/F_0_, and ETR. This axis reflects the photosynthetic efficiency gradient between healthy and stressed plants. The second canonical axis (Can2), accounting for 28.7% of the total variance, was primarily influenced by F_0_, which increases under stress conditions due to impaired reaction center activity and enhanced non-photochemical energy dissipation. Together, these axes distinguish genotypes with greater photochemical stability (e.g., UFVJM 08, UFVJM 18, and Rubissol), characterized by higher Fv/Fm and ETR values, from those exhibiting greater susceptibility (e.g., UFVJM 02, UFMG 03, and UFVJM 04). Since F_0_ did not correlate significantly with the other parameters, it was not considered a determining factor in the separation of genotypes.

The relationship between initial fluorescence (F_0_) and the ratio of variable to maximum fluorescence (Fv/Fm) is important for understanding plant stress responses and photosynthetic performance. Thus, the negative correlation between Fv/Fm and F_0_ in plants attacked by *B. somnulentella* suggests that damage by this pest to sweet potato leaves resulted in damage to the photosystem II (PSII) reaction centers. In plants under stress, the Fv/Fm ratio is generally inversely proportional to F_0_, i.e., an increase in F_0_ suggests that more energy is being dissipated as fluorescence rather than being used for photochemistry. Consequently, a reduction in Fv/Fm occurs [19,44]. *Cisaberoptus kenyae* Keifer (Acari: Eriophyidae) infestation of mango leaves reduced the Fv/Fm ratio, falling to the range of 25.15–28.43%, indicating stress and reduced photosynthetic capacity [27]. The combined analysis of Fv/Fm and F_0_ can be used as a diagnostic tool to differentiate between resistant and susceptible plant varieties to pest attack. Resistant varieties tend to maintain stable values of Fv/Fm and F_0_, whereas susceptible varieties exhibit significant changes in these parameters [2,17]. Following this line of thought, the genotypes UFVJM 08, UFVJM 18, and UFVJM 291 showed stable values for these two variables compared to the other genotypes, possibly indicating continued activity of open reaction centers in infested leaves. The negative correlation between F_0_ and Fm/F_0_ in healthy plants was expected, as they are interdependent parameters. The Fm/F_0_ ratio is an indirect indicator of the potential fluorescence amplification capacity of PSII. A higher Fm/F_0_ ratio, as observed for all sweet potato genotypes not attacked by *B. somnulentella*, indicates better photosynthetic performance and plant health. In contrast, a ratio less than three may indicate stress or damage to the photosynthetic apparatus [26,27].

## 4. Materials and Methods

### 4.1. Location and Conditions of the Experiments

The experiment was conducted in greenhouses in the Agronomy department of the Universidade Federal dos Vales do Jequitinhonha e Mucuri (UFVJM) in Diamantina, Minas Gerais, Brazil (18°10′ S and 43°30′ W, 1387 m). The genotypes were separated into two greenhouses: one contained the pest *B. somnulentella*, and the other did not.

The genotypes were planted in 10-L pots and kept in the greenhouses, where they were irrigated daily by overhead irrigation. Three sweet potato cuttings were transplanted into each pot. The soil was fertilized and amended according to the recommendations for pots [45]. The genotypes of sweet potato selected were Brazlândia branca, Rubissol, UFMG 03, UFVJM 01, 02, 04, 08, 18, 91, 291, and 526.

### 4.2. Chlorophyll a Fluorescence

Leaves of sweet potato were evaluated in pairs, from plants exposed to *B. somnulentella* and unexposed plants of the same genotype.

**Figure 3:**
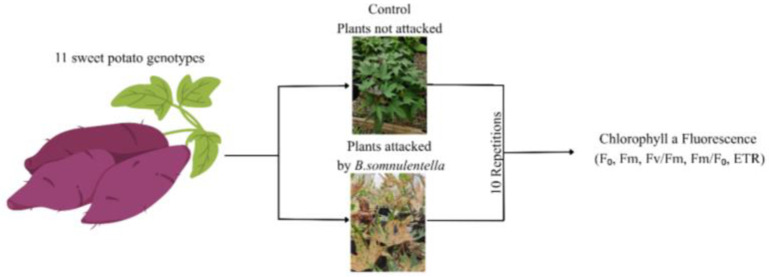
Schematic representation of the experimental setup used to evaluate *Ipomoea batatas* genotypes under *Bedellia somnulentella* (Lepidoptera: Bedelliidae) attack. Plants were grown in pots (three plants per pot) under greenhouse conditions. Eleven genotypes were evaluated under two conditions: non-attacked (control) and naturally attacked by the pest. One fully expanded leaf per plant was analyzed for chlorophyll *a* fluorescence parameters (F_0_, Fm, Fv/Fm, Fm/F_0_, ETR). Measurements were taken when approximately 40 ± 5% of the leaf area showed mining symptoms.

Eleven genotypes were chosen from the UFVJM germplasm bank based on representativeness of local diversity and agronomic interest. Because no validated resistant or susceptible standards to *B. somnulentella* were available for these genotypes, we adopted a screening approach: we assessed chlorophyll *a* fluorescence responses across a diverse set of genotypes under natural infestation. *Bedellia somnulentella* occurred naturally through a side opening in the greenhouse; however, the severity of damage was inspected and standardized before measurement. Only leaves showing approximately 40 ± 5% of the mined area were selected for fluorescence analysis. The percentage of leaf area damaged was quantified using the LeaFImage software (UFVJM), ensuring homogeneity in infestation levels among genotypes. Leaves with either minor or excessive damage were excluded to minimize variability (Figure 3).

Initial chlorophyll *a* parameter of minimum fluorescence (F_0_), maximum fluorescence (Fm), the ratio of maximum to minimum fluorescence (Fm/F_0_), the ratio between variable fluorescence and maximum chlorophyll *a* fluorescence (PSII potential quantum efficiency-Fv/Fm), and the electron transport rate (ETR-µmol electrons m^−2^ s^−1^) were evaluated for the genotypes 90 days after planting. Fluorescence measurements were performed with a portable PAM fluorometer (JUNIOR-PAM, Heinz Walz GmbH, Germany) on the middle third of the youngest fully expanded leaf of sweet potato plants, after 30 min of dark adaptation. The measuring light was modulated at 20 Hz, and saturating pulses of 0.3 s were applied at regular intervals to determine F_0_, Fm, Fv/Fm, and ETR parameters [35].

Genotypes that showed alterations in up to two photosynthetic parameters when attacked by *B. somnulentella* were classified as resistant, while those with variations exceeding this limit were considered susceptible.

### 4.3. Statistical Analysis

The experimental design was completely randomized (CRD) with eleven treatments [eleven with attack and eleven without attack (control)] and ten replicates, represented by the genotypes of sweet potato: Brazilândia Branca, Rubissol, UFMG 03, UFVJM 01, 02, 03, 04, 08, 18, 91, and 526.

The data were tested for homoscedasticity and normality of residuals prior to analysis. A one-way Analysis of Variance (ANOVA) was first performed to detect overall differences among treatments, followed by Student’s *t*-test (*p* ≤ 0.05) for pairwise comparisons between attacked and non-attacked plants within each genotype.

Correlation analysis was conducted with the software Sistemas para Análises Estatísticas e Genéticas (SAEG), version 9.1 [46] (Supplier: “Universidade Federal de Viçosa”). The correlation network procedure was performed using the qgraph package. The other analyses were performed using R software version 3.4.1, developed by the R Core Team.

A Principal Component Analysis (PCA) was performed using R software (version 3.4.1) to assess the multivariate relationships among chlorophyll *a* fluorescence parameters and to visualize the discrimination between attacked and non-attacked genotypes. The first and second principal components (Can1 and Can2) were used to construct the biplot shown in Figure 2. To improve visual interpretation, both axes were plotted using the same scale, and the two experimental groups were labeled as group 1 (non-attacked plants) and group 2 (attacked plants). Correlation network visualization was generated using the qgraph package in R, allowing the identification of positive and negative associations among variables.

## 5. Conclusions

This study demonstrates that *B. somnulentella* pest attack alters chlorophyll *a* fluorescence parameters in sweet potato genotypes, evidencing damage to photosystem II and reduced photosynthetic efficiency. Reductions in F_0_, Fm, Fv/Fm, Fm/F_0_ and ETR were consistently observed in several genotypes, particularly UFVJM 01, UFVJM 02, UFMG 03, UFVJM 04, UFVJM 91 and UFVJM 526, which showed higher sensitivity to infestation. In contrast, genotypes such as UFVJM 08, UFVJM 18, UFVJM 291, Brazlândia branca and Rubissol exhibited greater stability, maintaining more balanced fluorescence responses under pest pressure.

These results highlight the potential use of chlorophyll fluorescence as a rapid, non-destructive diagnostic tool for detecting physiological stress caused by leaf miners and for distinguishing resistant from susceptible genotypes. The identification of genotypes with higher tolerance provides an important basis for breeding programs and integrated pest management strategies, contributing to the development of more resilient and sustainable sweet potato production systems.

## Figures and Tables

**Table 1:** Mean values ± standard error of chlorophyll fluorescence parameters in different sweet potato (*Ipomoea batatas*) genotypes under non-attacked and insect-attacked conditions. Parameters evaluated: F_0_ (minimum fluorescence), Fm (maximum fluorescence), Fm/F_0_(Ratio of maximum to minimum fluorescence), Fv/Fm (maximum quantum efficiency of photosystem II), and ETR (electron transport rate). Means followed by the same letter within a row are not significantly different according to the *t*-test (*p* < 0.05). CV(%) S = coefficient of variation for non-attacked plants; CV(%) C = coefficient of variation for attacked plants.

Genotypes	Par.	Not Attacked	Attacked	CV(%) S	CV(%) C
UFVJM 01	F_0_	39.20 ± 1.60 a	23.90 ± 1.53 b	12.88	20.25
	Fm	93.50 ± 2.21 a	68.50 ± 2.53 a	31.16	11.70
	Fm/F_0_	3.91 ± 0.42 b	1.7 ± 0.82 a	12.81	18. 23
	Fv/Fm	0.66 ± 0.03 a	0.43 ± 0.02 b	15.15	15.84
	ETR	20.84 ± 1.11 a	18.66 ± 0.88 a	16.95	15.03
UFVJM 02	F_0_	41.90 ± 2.85 a	25.60 ± 2.01 b	21.51	24.86
	Fm	109.30 ± 9.74 a	76.20 ± 4.72 a	28.18	19.60
	Fm/F_0_	4.26 ± 0.52 b	1.81 ± 0.78 a	11.21	13.09
	Fv/Fm	0.72 ± 0.02 a	0.45 ± 0.02 b	10.12	18.66
	ETR	23.29 ± 1.31 a	15.14 ± 1.12 b	17.86	23.46
UFMG 03	F_0_	42.10 ± 8.85 a	22.00 ± 1.97 b	21.43	28.35
	Fm	93.30 ± 5.68 a	66.3 ± 3.36 a	19.26	16.06
	Fm/F_0_	4.24 ± 0.54 b	1.57 ± 0.62 a	13.7	29.49
	Fv/Fm	0.72 ± 0.02 a	0.35 ± 0.04 b	12.64	38.52
	ETR	20.32 ± 0.84 a	14.94 ± 0.81 a	13.18	17.21
UFVJM 04	F_0_	35.60 ± 1.84 a	21.60 ± 1.10 b	26.34	29.13
	Fm	111.40 ± 3.33 a	69.90 ± 2.97 b	9.47	13.46
	Fm/F_0_	5.15 ± 0.11 b	1.96 ± 0.38 a	13.78	19.38
	Fv/Fm	0.75 ± 0.03 a	0.51 ± 0.02 b	13.40	16.15
	ETR	19.38 ± 1.24 a	13.37 ± 0.99 a	20.26	23.54
UFVJM 08	F_0_	38.10 ± 3.53 a	23.90 ± 2.21 a	29.37	29.29
	Fm	108.60 ± 8.92 a	82.10 ± 5.51 a	25.99	21.26
	Fm/F_0_	4.54 ± 0.65 b	2.15 ± 0.73 a	18.91	23.95
	Fv/Fm	0.73 ± 0.06 a	0.53 ± 0.04 a	28.63	27.52
	ETR	21.01 ± 1.72 a	18.37 ± 0.86 a	25.92	14.91
UFVJM 18	F_0_	34.70 ± 2.78 a	21.40 ± 2.05 a	25.27	30.30
	Fm	91.0 ± 5.85 a	59.40 ± 3.08 b	20.34	16.44
	Fm/F_0_	4.25 ± 0.42 b	1.71 ± 0.65 a	13.05	18.01
	Fv/Fm	0.64 ± 0.03 a	0.40 ± 0.05 a	16.69	38.99
	ETR	17.75 ± 1.14 a	13.96 ± 0.49 a	20.42	11.13
UFVJM 91	F_0_	36.80 ± 2.81 a	20.90 ± 1.88 b	24.16	28.57
	Fm	90.10 ± 10.87 a	68.0 ± 5.99 a	38.18	27.82
	Fm/F_0_	4.31 ± 0.54 b	1.85 ± 0.73 a	19.79	19.45
	Fv/Fm	0.68 ± 0.03 a	0.43 ± 0.03 b	13.96	26.62
	ETR	19.43 ± 0.83 a	16.35 ± 1.27 a	13.64	24.69
UFVJM 291	F_0_	41.3 ± 2.53 a	24.90 ± 3.94 a	19.44	50.14
	Fm	110.20 ± 6.10 a	70.60 ± 2.62 b	17.52	11.78
	Fm/F_0_	4.43 ± 0.64 b	1.71 ± 0.69 a	17.34	21.35
	Fv/Fm	0.51 ± 0.01 a	0.41 ± 0.02 a	11.70	20.49
	ETR	20.20 ± 1.59 a	16.26 ± 0.98 a	24.93	19.23
UFVJM 526	F_0_	39.50 ± 2.23 a	21.30 ± 2.51 b	17.87	37.37
	Fm	119.70 ± 4.10 a	71.30 ± 4.35 b	13.20	19.30
	Fm/F_0_	5.62 ± 1.02 b	1.81 ± 7.38 a	21.67	36.42
	Fv/Fm	0.55 ± 0.02 a	0.42 ± 0.03 a	11.60	27.01
	ETR	19.68 ± 0.63 a	15.31 ± 0.69 b	10.87	14,42
Brazlândia branca	F_0_	34.10 ± 1.10 a	17.60 ± 2.33 b	18.52	42.03
	Fm	95.50 ± 7.81 a	66.90 ± 4.59 a	25.87	21.72
	Fm/F_0_	5.43 ± 0.67 b	1.96 ± 0.91 a	20.87	30.35
	Fv/Fm	0.69 ± 0.03 a	0.48 ± 0.03 a	17.25	20.33
	ETR	23.79 ± 1.28 a	17.93 ± 0.64 a	17.07	11.35
Rubissol	F_0_	30.40 ± 1.67 a	16.10 ± 1.51 b	17.28	29.78
	Fm	100.60 ± 6.91 a	69.30 ± 2.09 a	21.74	9.57
	Fm/F_0_	6.25 ± 0.56 b	2.28 ± 0.78 a	10.99	34.21
	Fv/Fm	0.69 ± 0.02 a	0.52 ± 0.02 a	10.00	16.83
	ETR	22.35 ± 1.48 a	17.14 ± 0.72 a	21.07	13.45

## Data Availability

Data can be provided upon request from the first author Maria J. S. Cabral.
